# Efficiently passing messages in distributed spiking neural network simulation

**DOI:** 10.3389/fncom.2013.00077

**Published:** 2013-06-10

**Authors:** Corey M. Thibeault, Kirill Minkovich, Michael J. O'Brien, Frederick C. Harris, Narayan Srinivasa

**Affiliations:** ^1^Center for Neural and Emergent Systems, Information and System Sciences Laboratory, HRL Laboratories LLC.Malibu, CA, USA; ^2^Department of Electrical and Biomedical Engineering, The University of NevadaReno, NV, USA; ^3^Department of Computer Science and Engineering, The University of NevadaReno, NV, USA; ^4^Department of Mathematics, University of California at Los AngelesLos Angeles, CA, USA

**Keywords:** parallel spiking neuron simulation, neural networks, parallel simulation, distributed computing, distributed message passing

## Abstract

Efficiently passing spiking messages in a neural model is an important aspect of high-performance simulation. As the scale of networks has increased so has the size of the computing systems required to simulate them. In addition, the information exchange of these resources has become more of an impediment to performance. In this paper we explore spike message passing using different mechanisms provided by the Message Passing Interface (MPI). A specific implementation, MVAPICH, designed for high-performance clusters with Infiniband hardware is employed. The focus is on providing information about these mechanisms for users of commodity high-performance spiking simulators. In addition, a novel hybrid method for spike exchange was implemented and benchmarked.

## 1. Introduction

The highly distributed nature of the animal nervous system presents a unique challenge in theoretical and computational modeling of neurobiology. Whether these models are intended to provide a better understanding of biological function or to build more intelligent agents, the comparatively limited parallelization inherent in all modern computing architectures must be overcome to achieve models that accurately represent the highly parallel nature of biology. The current computing and software paradigms have prevented truly scalable neural models that can faithfully simulate biology in reasonable amounts of time. In addition, a compromise between biological realism and performance must be made. This is a concession that is often unacceptable to the overall performance of the task.

There are two major steps in simulating the nervous system: incrementally solving the governing equations and communicating the results to other parts of the system. We previously presented ways of improving the performance of the former by parallelizing the computations on clusters of General Purpose Graphical Processing Units (GPGPU) (Thibeault, 2012). The purpose of this work is to demonstrate where the spike communication can be optimized on generic high-performance computing architectures.

The effort to efficiently simulate spiking neural networks has a long history that spans hardware implementations (VLSI and FPGA) and the more popular highly distributed compute cluster implementations. Although hardware options are increasing in popularity with projects like SPINNAKER (Furber et al., 2012) and SyNAPSE (Merolla et al., 2011; Srinivasa and Cruz-Albrecht, 2012), they still cannot compete with the practicality and flexibility of generalized simulators. Even the aforementioned hardware options are generally supported by high-performance distributed simulation environments.

Recently, Hines et al. (2011) explored several different spike exchange methods on an IBM Blue Gene/P (BG/P) cluster and concluded that point-to-point communication using the built-in standard Message Passing Interface (MPI) non-blocking MPI_Isend was the worst performing method. Of the top performing methods of that work, the MPI collective routine, MPI_Allgather, was among the best; often with simulation times comparable to the BG/P specific direct memory access routines.

Hardware such as the BG/P provide unprecedented performance per watt but comes with a price point that can be out of reach to most computational neuroscientists. Because of this, commodity clusters using Commercial Off-The-Shelf (COTS) components are more prevalent in research labs. With the availability of GPUs, the architecture of COTS clusters has changed considerably. Unlike the BG/P architecture were there can be over 100,000 processors linked together, GPU based COTS clusters have much higher processing capabilities at the single node (computer within the cluster) level. These then share a common communication link. The dense parallelization available on a single node allows for a much larger number of computations but results in a communication bottleneck as more information must be shared between nodes.

Morrison et al. (2005) presented a generic architecture for distributed neural computation in which they contend that the amount of time spent in communication is small compared to the amount of time required to update the neurons. This appears to be a reasonable statement so long as the number of compute nodes is small. However, as both the number of compute nodes and the number of neurons simulated increases the amount of time spent in communication becomes significant.

In this paper we explore how the cost of spike message passing can be reduced. To begin with, several different communication mechanisms provided by the MPI standard are explored on a COTS computing cluster using both Infiniband and Ethernet hardware backends. This is in contrast to the work of Hines et al. (2011) which focused on the BG/P architecture. We then present a novel hybrid method that combines two spike exchange schemes to reduce the maximum amount of information sent between compute nodes. These results not only benefit users of high-performance spiking neural network simulators but also the neuromorphic engineering community.

## 2. Methods

When distributing the network simulation, different portions of the model are simulated by separate computers in parallel. The neurons in the model are integrated at each iteration, and the spiking information is sent to all the neurons connected to those that fired. Ideally, when performing this parallelization, the computational cost of the mathematical integration and synaptic computations is balanced with cost of communicating information between nodes. Historically, as mentioned above, the communication time was significantly lower than the compute time. With the introduction of higher-performance architectures such as General Purpose Graphical Processing Units (GPGPU) and specialized neural hardware systems, this is no longer the case. However, the manner by which spiking information is sent has not changed.

Almost all hardware and software simulation environments use a variant of address event representation (AER) (Boahen, 2000). The simplest and most efficient implementation of AER sends a firing neuron's unique identification number to all of the nodes containing any of that neuron's targets. In general, all of the neurons that fire during the current iteration can be collected and sent as a single packet to all of the connected nodes.

As the number of neurons that fired increases, the size of the data packets correspondingly increase. In this case, the time spent in communication is a direct correlation to the number of neurons that fired. Similarly, as the number of compute nodes increases so does the number of packets that need to be sent. In some cases, for both software and hardware based systems, this can prevent scaling up to desirable model sizes.

### 2.1. Dummy neurons

**Figure 1:**
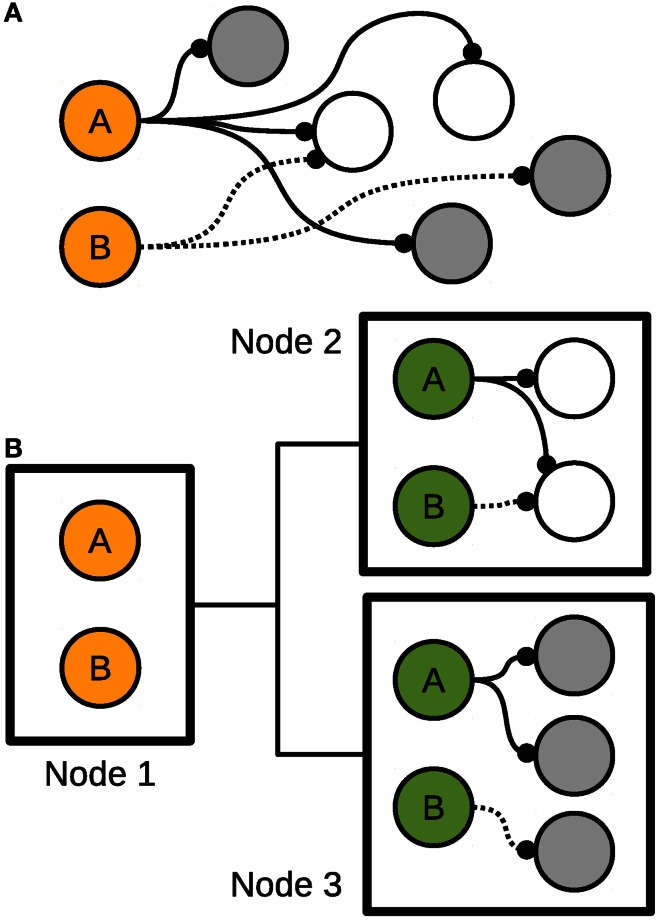
**Dummy neurons. (A)** A sample network. **(B)** Distribution of the sample network among three nodes.

HRLSim (Thibeault, 2012) uses the concept of dummy neurons to not only reduce the amount of information distributed for a spike event but also the complexity of updating the synaptic weights. Dummy neurons are essentially copies of pre-synaptic neurons that are located on remote compute nodes. These neuron copies receive the spiking information from the remote neuron and then relay that to all of the locally connected post-synaptic neurons. In addition, the pre-synaptic information is computed at the dummy neurons locally, rather than on the remote node. This scheme is illustrated in Figure 1.

### 2.2. Rate independent message passing

In Thibeault et al. (2011) the spiking information between nodes was encoded as single bits within a packet. Essentially, each output neuron is represented by a bit, where a “1” indicates that neuron fired and a “0” indicates it did not. This bit-packed representation was used to simplify some of the GPU computations but abused the spike message passing by sending packets that are larger than necessary. Here we propose a method of combining AER with that bit-packed representation referred to here as the hybrid message passing scheme. The key to this method is that the transition to bit-packing is only done when firing rates are high enough that it will reduce packet sizes. In addition, this is only performed between the nodes with neurons that satisfy the firing rate requirement. The novelty of the hybrid message scheme lies in its deterministic upper bound performance, flexibility to neuron firing rates and scalability greater than traditional message passing methods.

**Figure 2:**
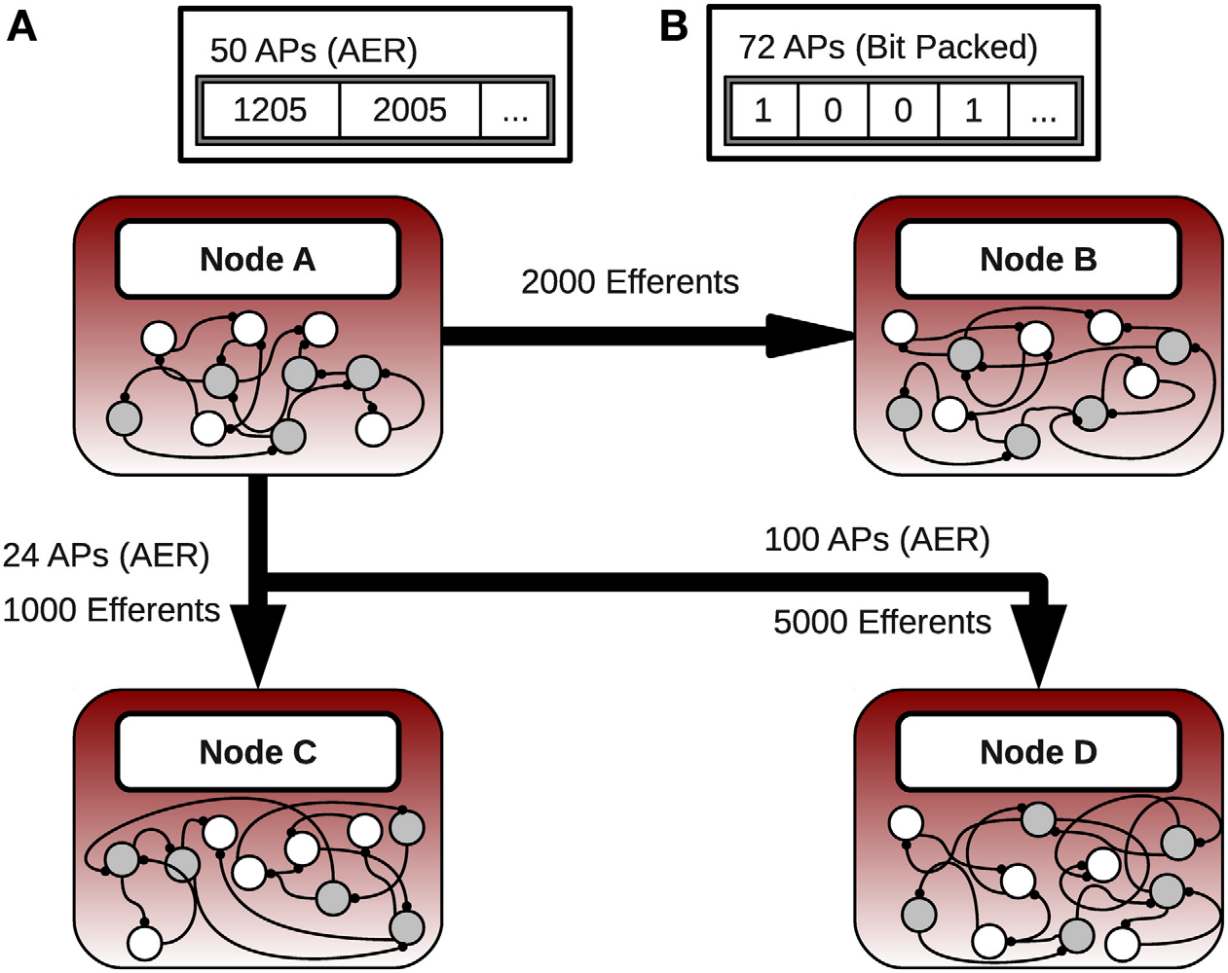
**Hybrid message passing. (A)** The number of neurons that fired are below the transition threshold. **(B)** When the number of neurons increases past the threshold the simulator automatically switches into the bit-packing mode.

The network in Figure 2 illustrates the use of the hybrid scheme. There are four compute nodes, each simulating a group of neurons. Consider node A, which has 2000 neurons with projections to node B, 1000 neurons with projections to node C, and 5000 neurons with projections to node D. The maximum communication cost associated with transferring action potentials between the populations and the remote nodes is a function of these population sizes as is the theoretical transition point.

The transition point between the protocols in the hybrid scheme is where it is computationally cheaper to represent the neurons in a bit-packed notation compared to traditional AER. Suppose that on node A at a particular iteration, 50 neurons connected to B fire an action potential, 24 neurons connected to C fire, and 100 neurons connected to D fire. In this case the AER scheme is used to communicate between all nodes. If instead 72 neurons with efferents on B fire, and everything else stayed the same, then the bit-packed scheme is used only between nodes A and B. In this case, only 63 integers would have to be transferred instead of the 72 with the AER scheme (we are assuming that integers are 32-bits wide throughout this paper).

A single byte at the beginning of each packet is use to facilitate the dynamic switching between message packing schemes. For the AER scheme this header byte indicates the total number of firings contained in the current message. For the bit-packed scheme this will be a negative value signaling the receiving node to process the packet as such.

For example the physiologically realistic action selection model of Thibeault (2012) is a case where the rate-independent message passing scheme could have a significant performance impact. The network consists of three micro-channels of the rodent basal ganglia using 576 neurons with an integration time-step of 1 ms. For this example, it is assumed that the 192 neurons in the external segment of the Globus Pallidus (GPe) have output projections whose spiking information must be passed to another node. Physiologically the rodent GPe has a basal level of activity around 30 Hz (Humphries et al., 2007), which is a level where other message passing schemes show performance degradation.

**Figure 3:**
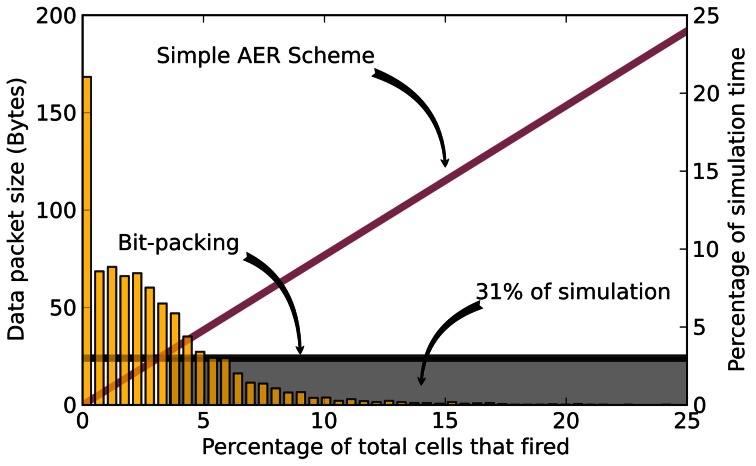
**Example spike generation for a 5 s simulation of the GPe region of the network model of Thibeault (2012)**. The packet size for the AER scheme (purple line) and bit-packing scheme (black line) are shown. The histogram corresponds to the percentage of simulation time where the GPe region fired that percentage of cells during an integration step. The gray box highlights the region where the hybrid method would use the bit-packing scheme (31% of the time).

It takes 18 integers to encode all 576 neurons, which is equivalent to encoding 3.125% of the total outputs with AER. Figure 3 illustrates the amount of simulation time spent for the different rates of spiking activity over a 5 second simulation of basal activity. The results show that 31% of the simulation time is spent in the region were more than 18 neurons fire.

### 2.3. Benchmark experiments

Here we explore two different aspects of spike exchange with MPI on general computing architectures. The first, is the type of communication mechanism. The performance between two peer-to-peer, blocking and non-blocking, and one collective communication method, all to all variable (alltoallv), using the included MPI functionality were analyzed to determine if one demonstrated a clear benefit. These experiments were completed for both Infiniband communication fabric and standard Ethernet using the AER method.

The blocking communication is accomplished with separate calls to MPI_Isend and MPI_Recv. Whereas the non-blocking scheme uses MPI_Isend combined with MPI_Irecv. This method allows the underlying communication to be handled by the MPI threads. The alltoallv method uses a single call to MPI_Alltoallv to complete the spike exchange. A disadvantage to MPI collective methods is that they block processing so no other computations can be performed while spikes are exchanged. Here, in order to allow for communication and the spike computations to occur in parallel, the spike exchanges for collective communication were threaded.

The second aspect explored was the benefit of the hybrid message passing scheme as well as the optimal pivot point. The pivot point is a multiplier used to determine where the transition point occurs in relation to the number of efferent connections to a remote node. For a pair of nodes A and B, let *N* represent the number of node A neurons that project onto B. For a given time step, denote the number of those *N* neurons that spike as *S*, and let *F* be the hybrid threshold number for which when *S* ≤ F, AER is used, and for *S* > F, bit-packing is used. Then, we define the multiplicative pivot point *P* by:

(1) $$F=P\frac{N}{32},$$

where 32 is the number of bits in an integer. *P* is the parameter considered in the following benchmarks. Note that *P* = 0 indicates that bit-packing is always used and corresponds to the horizontal line of Figure 3, *P* = 32 would mean the AER message passing scheme is used exclusively and corresponds to the diagonal line of Figure 3, and *P* = 1 would mean the hybrid scheme is used and corresponds to the intersection of the the horizontal and diagonal lines of Figure 3. Currently only the non-blocking and alltoallv mechanisms have the option of bit-packing and since the initial performance for the non-blocking method was better, it was chosen for the hybrid message passing experiments. During these, pivot points of *P* ∈ {0, 1, 2, 3, 10, 20, 32} were used.

For both the communication mechanism experiments and hybrid method experiments, two different types of networks were simulated, strong scaling and weak scaling. The networks are summarized in Tables 1, 2. The strong scaling experiments explore networks of the same size distributed over a larger number of compute nodes. In the weak scaling experiments the size of the network increases in direct correlation with the number of nodes. Only communication time is measured in the benchmarks and each trial was run three times, with the lowest trial time reported here.

**Table 1:** **Strong scaling experiments**.

**Nodes**	**Neurons**	**Connections per Neuron**
8	2,000,000	1000
16	2,000,000	1000
32	2,000,000	1000
64	2,000,000	1000
96	2,000,000	1000
8	250,000	10,000
16	250,000	10,000
32	250,000	10,000
64	250,000	10,000
96	250,000	10,000

**Table 2:** **Weak scaling experiments**.

**Nodes**	**Neurons**	**Connections per Neuron**
8	2,000,000	1000
16	4,000,000	1000
32	8,000,000	1000
64	16,000,000	1000
96	24,000,000	1000
8	250,000	10,000
16	500,000	10,000
32	1,000,000	10,000
64	2,000,000	10,000
96	3,000,000	10,000

### 2.4. Hardware

The Infiniband fabric is a hardware level communication system specifically designed for high-performance applications. It offers low-latency and high-bandwidth over short distances. In contrast, Ethernet hardware is a ubiquitous technology found on most modern computing architectures. It is primarily used for local-area connections and includes the physical and data link layers of the Open System Interconnection model. Although, in high-performance systems hardware communication fabrics like Infiniband are more prevalent, evaluating the lower bandwidth and lower latency mechanisms is important for both remote applications (i.e., robotics), as well as inexpensive high-performance clusters.

The benchmarks presented here were completed on a cluster of 92 compute nodes, each with two Intel Xeon E5520 2.27 GHz CPUs and two NVIDIA Tesla C1060 cards, with Infiniband and Gigabit Ethernet communication backends.

### 2.5. Test suite

**Figure 4:**
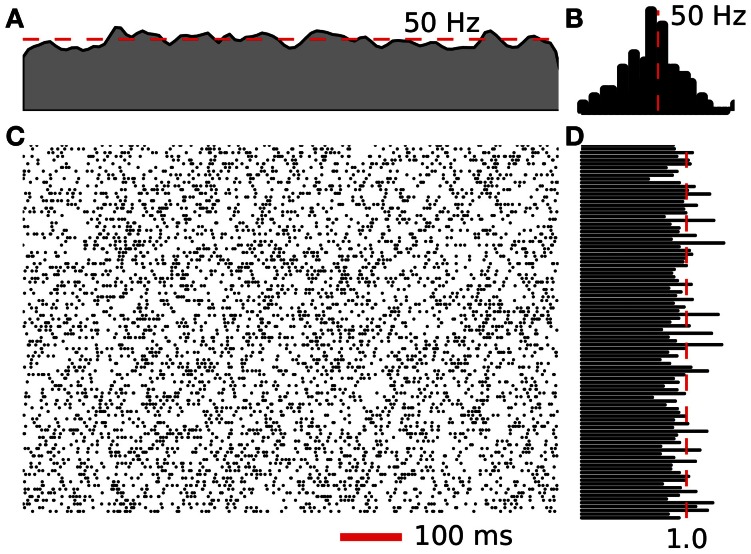
**Example activity of 200 neurons from a 50 Hz Poisson network. (A)** Fire rate of the network calculated using a Gaussian window. **(B)** Neuron spike frequency histogram. **(C)** Raster plot of spiking activity for 1 s. **(D)** Coefficient of variation for the 200 neurons displayed in **(C)**.

A software suite was developed to facilitate the benchmarks. The suite consisted of C++ implementations of network generation, neuron spike generation, and spike exchange, along with Python modules for job submission, results analysis and plotting. The network is split randomly with each node simulating the same number of neurons. The connections are randomly selected from a uniform distribution. The neural activity is generated by a Poisson random point process with the center at the target frequency. Figure 4 illustrates the activity and statistics for 200 neurons of the test network. Four target frequencies were used, 10, 30, 50, and 80 Hz. The spike exchange was then controlled by one of the three mechanisms described above. Simulations are run for a duration of 1 s and a minimum of 3 trials were completed. However, in some cases, where the results were inconsistent, more trials were conducted. The test suite was compiled with the MVAPICH 1.7 library from the Network-Based Computing Laboratory at The Ohio State University.

## 3. Results

### 3.1. Communication methods

#### 3.1.1. Infiniband

**Figure 5:**
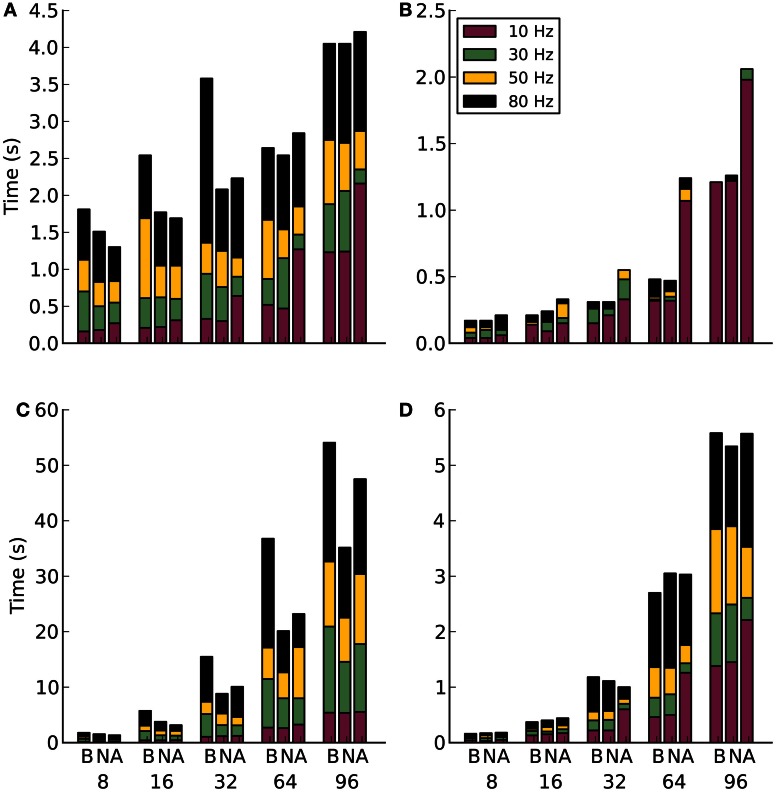
**Results for communication method experiments on Infiniband**. Along the x-axis are the different communication methods for the different number of compute nodes. The letters correspond to B-blocking, N-non-blocking, A-alltoallv. The subplots are: **(A)** Strong scaling for 1000 efferent synapses. **(B)** Strong scaling for 10,000 efferent synapses. **(C)** Weak scaling for 1000 efferent synapses. **(D)** Weak scaling for 10,000 efferent synapses.

The strong scaling results shown in Figures 5A,B reveal an interesting trend in the cost of increasing the distribution of a network. In theory, as a network is distributed over more compute nodes, the performance will increase. This is an effect of reducing the amount of computational work required by each node. However, as illustrated here, the communication cost rises with a corresponding increase in compute nodes. Eventually the communication cost becomes greater than the parallelization benefit. This is an important consideration for balancing the number of neurons per node with the number of nodes. Unless the network activity and the number of compute nodes is low, the simulations would be unable to run in real-time. The real-time measure is particularly important for embodied modeling where simulations need to run fast enough for control of the agent.

Another interesting trend can be seen in the blocking communication results of Figure 5A. At first these were assumed to be anomalies. However, after running 12 extra simulations for 8, 16, and 32 nodes at both 50 and 80 Hz firing rates the results stayed consistent. It is still unclear why there is a drop in simulation time at 64 nodes compared to 8, 16, and 32.

The benefit of dummy neurons is illustrated in Figure 5B. There is a clear penalty to encoding more spike messages, however, it is not dependent on firing as in Figure 5A. This is likely due to the smaller number of neurons.

The weak scaling experiments shown in Figures 5C,D show how an increase in both neurons and nodes can affect the overall performance. However, the correlation between the two follows an exponential trend rather than a linear one.

Overall, on Infiniband hardware, the choice of communication method seems to favor the non-blocking method. This is slightly surprising in the context of early hardware characterizations (not shown), where the blocking and non-blocking methods appeared to be identical. The non-uniform nature of the Poisson network is the likely explanation for the difference. The non-blocking code allows message processing to happen out of order, favoring those that are sent earlier. There is obviously less time wasted waiting for messages to arrive in order.

#### 3.1.2. Ethernet

**Figure 6:**
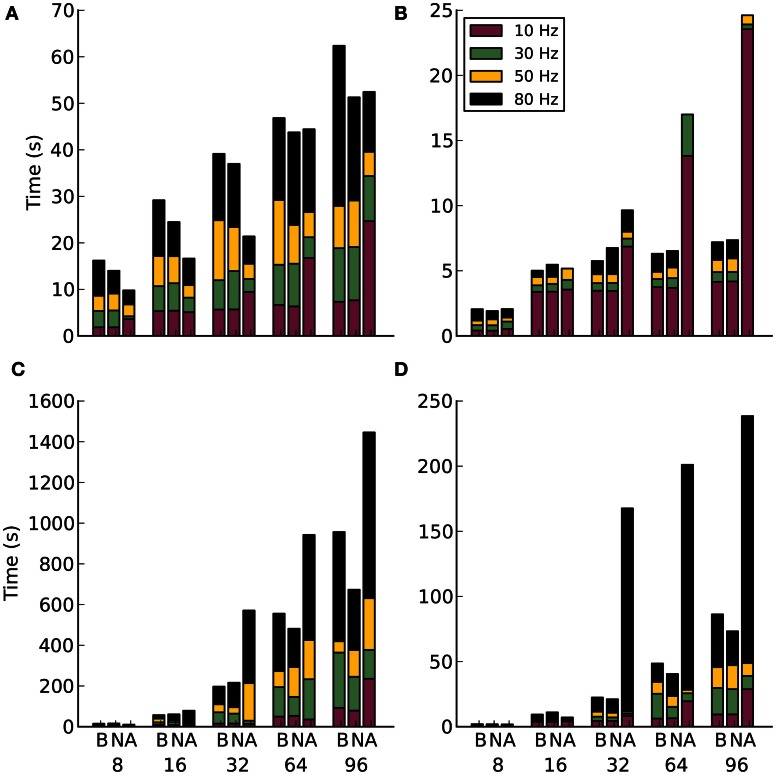
**Results for communication method experiments on Ethernet**. Along the x-axis are the different communication methods for the different number of compute nodes. The letters correspond to B-blocking, N-non-blocking, A-alltoallv. The subplots are: **(A)** Strong scaling for 1000 efferent synapses. **(B)** Strong scaling for 10,000 efferent synapses. **(C)** Weak scaling for 1000 efferent synapses. **(D)** Weak scaling for 10,000 efferent synapses.

The strong scaling experiments on Ethernet hardware in Figure 6A show a similar increase to that seen on Infiniband. However, the alltoallv method shows an advantage for higher rates of activity and lower number of nodes. Why this happens is again unclear, as is the large increase in communication time at 64 and 96 nodes for the alltoallv method. When the number of efferent connections is increased to 10,000, Figure 6B, a surprising plateau in the blocking and non-blocking schemes emerges between 16 and 96 nodes. The alltoallv scheme however, continues to trend upwards.

For the weak scaling experiments, Figures 6C,D, the alltoallv scheme actually performs considerably worse than the other two methods. With the non-blocking scheme generally demonstrating better timings throughout the weak scaling experiments.

### 3.2. Hybrid message passing

In the bit-packing experiments on Infiniband hardware an optimal pivot point for a given experiment was not found despite thorough analysis (not shown). Although on Ethernet hardware some trends toward an optimal pivot point did appear, it was still difficult to predict where that point would lie for a generic network (not shown). Optimally, the pivot point would be adapted dynamically, during a simulation. This will result in improved communication performance as the simulation progresses and is something that will be explored in future work.

**Figure 7:**
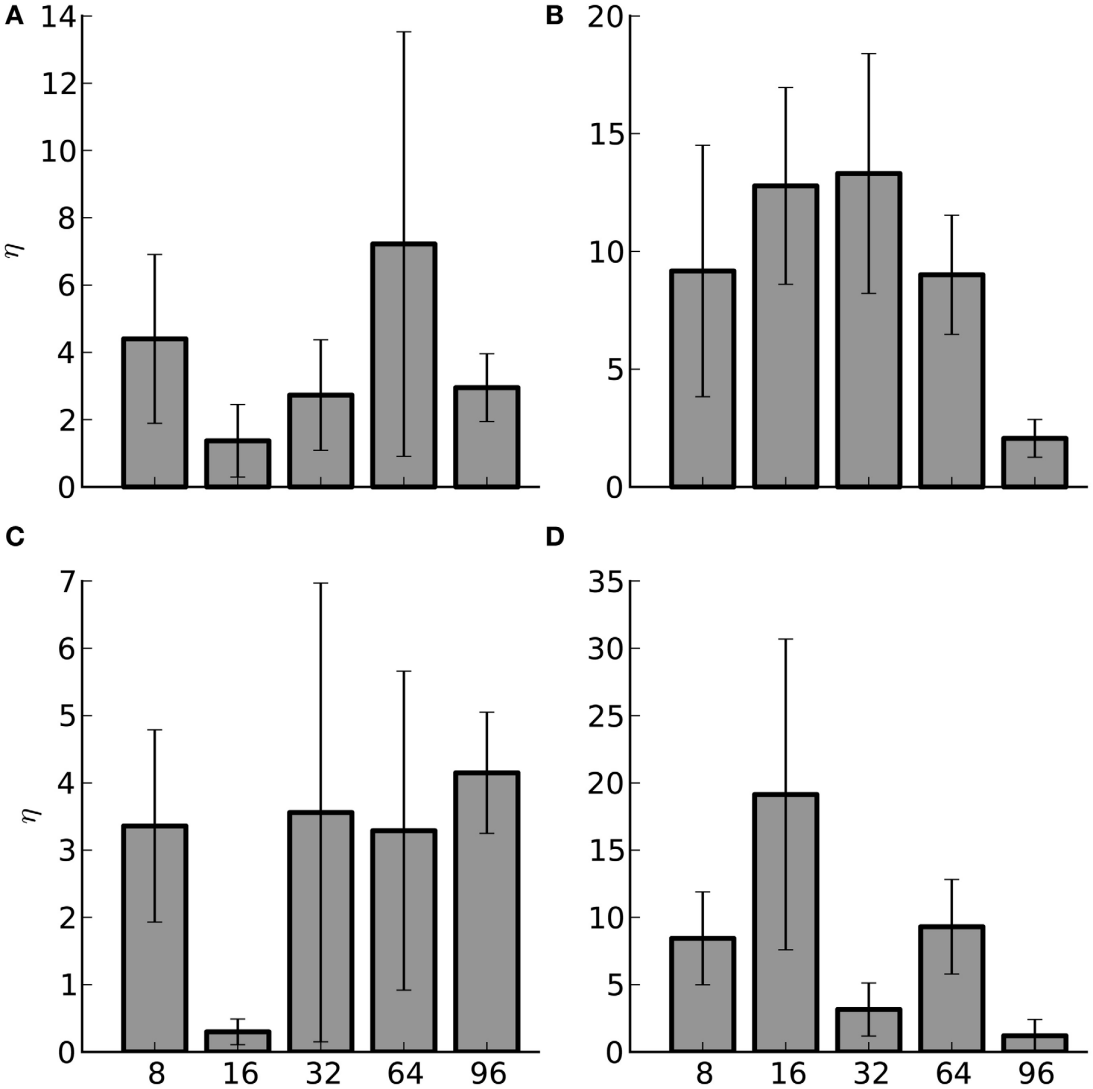
**Average percent improvement between the hybrid method and the AER method (η) and the standard error of the mean (SEM) for Infiniband hardware. The number of nodes are listed along the x-axis**. The subplots are: **(A)** Strong scaling for 1000 efferent synapses. **(B)** Strong scaling for 10,000 efferent synapses. **(C)** Weak scaling for 1000 efferent synapses. **(D)** Weak scaling for 10,000 efferent synapses.

Despite the difficulty in selecting the optimal pivot point, in 86% of the cases, 69 out of 80, using the hybrid method shows an improvement over the AER method alone. The improvement is illustrated in Figure 7, where the average percent change, η, between the hybrid method at the optimal pivot point and the AER method is computed for each set of experiments at each of the compute node quantities. The average was taken over the four target activity rates since most large-scale experimental networks rarely maintain a uniform fire rate. Despite the fact the gains from the hybrid method are modest there is still a benefit to using it.

**Figure 8:**
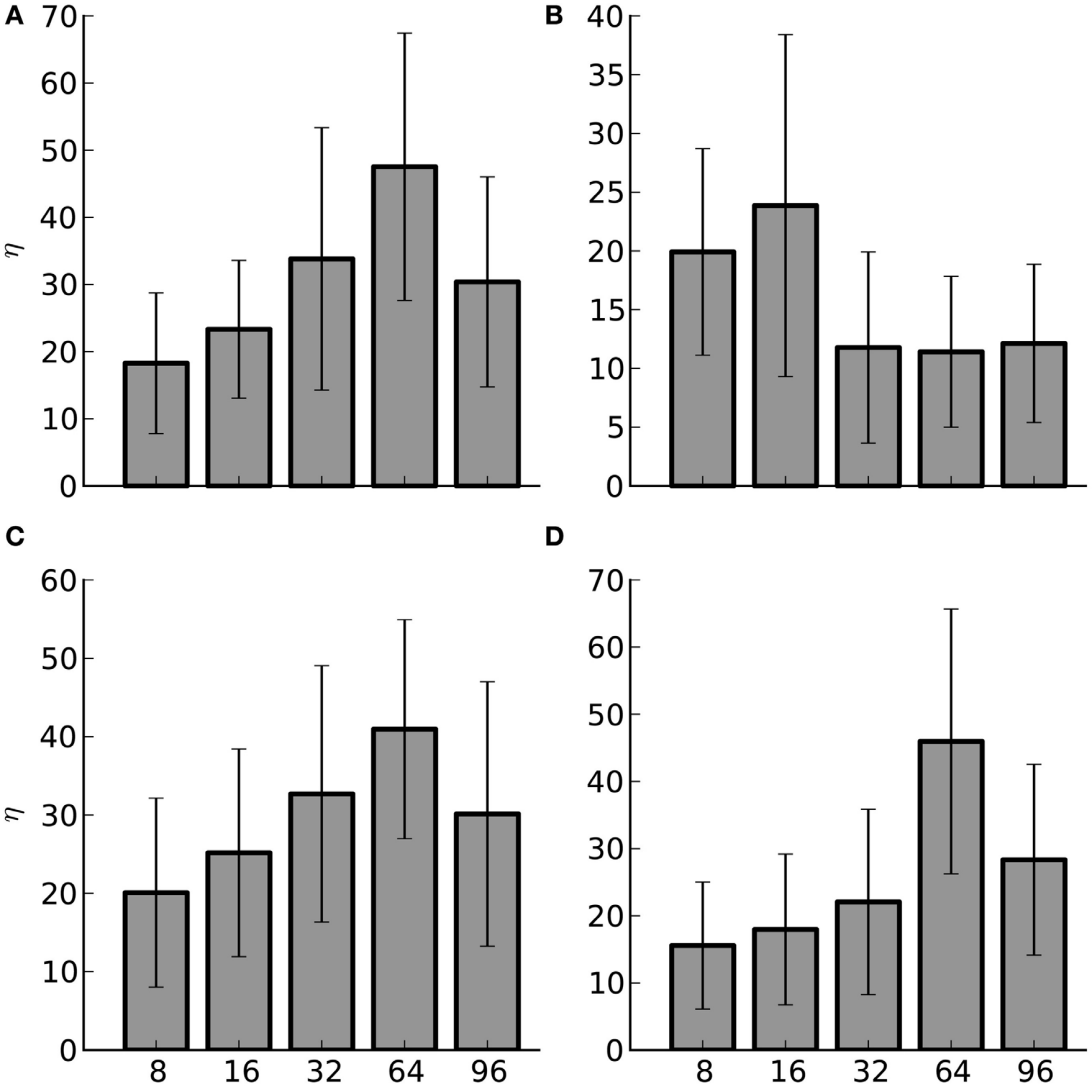
**Average percent improvement between the hybrid method and the AER method (η) and the SEM for Ethernet hardware**. The number of nodes are listed along the x-axis. The subplots are: **(A)** Strong scaling for 1000 efferent synapses. **(B)** Strong scaling for 10,000 efferent synapses. **(C)** Weak scaling for 1000 efferent synapses. **(D)** Weak scaling for 10,000 efferent synapses.

On the Ethernet hardware the hybrid method offers substantial performance improvement compared to Infiniband. These experiments resulted in 96% of the simulations, 77 out of 80, showing a benefit to employing the hybrid method. Figure 8 illustrates the average percent improvement between the four different rates for the experiments; again this was at the optimal pivot point for each of the experiments. In general, using the hybrid method resulted in 11–47% improvement. For large-scale systems this is a significant gain. In addition to Ethernet hardware this result is important for hardware based spiking neural networks. In these, the available communication fabric between compute elements is generally not as powerful as that provided by Infiniband. Employing the hybrid method in these instances could offer important performance benefits.

## 4. Discussion

Past research in spike exchange methods has been sparse. Many groups present a single communication mechanism with minimal justification for its selection. The majority of general simulation environments use the point-to-point blocking mechanisms in MPI (Wilson et al., 2001; Morrison et al., 2005; Pecevski et al., 2009). Morrison et al. (2005) combined that with the Complete Pairwise EXchange (CPEX) algorithm. At the time this was selected based on the assumption that it was more robust. However, it was later stated that the collective, MPI_Allgather, was more efficient on certain hardware (Eppler et al., 2007); benchmarks were not presented to support that claim. PCSIM also uses blocking communication with the CPEX algorithm (Pecevski et al., 2009).

The NEURON simulation environment is one of the few that use the collective *MPI_Allgather* as opposed to the point-to-point methods (Migliore et al., 2006). This decision is based on the simplicity of the implementation and that the performance of NEURON is dominated by the more complex models that are its niche. However, the use of NEURON on the BG/P Supercomputer was the motivating factor for work presented in Hines et al. (2011). This is the most current analysis of different spike-exchange methods but is unfortunately specific to the BG/P hardware. The work presented here is the first analysis aimed at the COTS hardware more readily available to the computational neuroscience community.

### 4.1. Choosing a communication mechanism

Selecting a spike exchange method is still a difficult problem. The type of hardware as well as the configuration can create situations where one method clearly outperforms. The results of this work suggest that a safe pick for COTS architectures would be the non-blocking point-to-point communication methods. This is contradictory to the results found for Infiniband backend in Eppler et al. (2007), and the BG/P hardware in Hines et al. (2011). This analysis will need to be repeated as new hardware as well as more optimized communication methods are released. In the future we hope to package and release this work to provide an automated mechanism for selecting the highest performing communication method for a given hardware setup.

#### 4.1.1. Why not alltoall?

The results of early communication characterizations suggested that for truly large-scale simulations, using the basic alltoall mechanism, rather than alltoallv, would offer much higher performance (not shown). The motivation behind alltoall is to reduce code complexity while allowing developers of the MPI middleware to optimize the functions at the device level. In MVAPICH2 alltoall in particular has been the focus of significant optimizations on Infiniband (Sur et al., 2005).

The alltoall collective requires that all nodes send the exact same amount of information to each node in the simulation. Message packets must be a fixed size and the overflow of those must be handled by a separate mechanism. Early testing with this method in the neural simulator presented in Thibeault (2012) resulting in performance that was much worse than the methods presented here. However, recent tests (not shown) suggest that the alltoall method may be beneficial for networks using greater than 96 nodes. In the future we plan to expand the original code base and rerun the benchmarks completed here to see if there is in fact a niche for the alltoall method.

Another possible benefit of this method that was not tested, is the reduced overhead in creating the spiking messages. In the methods benchmarked here each node keeps a local buffer that is filled with spiking information. These are contiguous in memory and require some form of locking to prevent multiple compute threads writing to the same location. With the alltoall fixed message size scheme, each thread can be assigned a unique section of the buffer; enforcing mutual exclusion. This would allow the removal of thread-blocking which may provide another point of optimization. This concept will be tested in the future.

### 4.2. Hybrid message passing

With software simulation environments, there is generally a computational cost associated with packing the spike messages. In most cases it is insignificant or can be reduced by using GPGPU's, which are designed for just such parallel tasks. The hybrid spike passing scheme has already proven effective in large-scale cluster based neural simulations by HRL (Thibeault, 2012). It is important to emphasis in these large-scale simulations that the overall activity of the network does not need to be high in order for the hybrid message passing to be effective. The method will only switch to bit-packing when the activity of the neurons connected to a particular node are above the threshold set by the pivot point. This effectively balances the communication load throughout the cluster.

In addition to large-scale simulations, this technique can also improve the performance of communication between neuromorphic architectures. These have traditionally used AER schemes. Take for example SpiNNaker (Khan et al., 2008), which was designed to use an AER communication to simulate a neural network with a firing rate of 10 Hz. The final hardware was theoretically able to simulate networks firing up to 77.5 Hz (Navaridas et al., 2009). Once the network is firing above this rate, either spikes would have to be dropped or the whole system slowed down. The problem is that in biological systems, even though the firing rate is on average low, there are times and regions, in which the firing rate goes beyond 100 Hz. Using the hybrid encoding scheme would allow for scaling to any firing rate.

Finally, as outline above, the existing simulators available to researchers use either the AER or the bit-packing scheme. In comparing the two methods it was found that for Infiniband hardware exclusively using the bit-packing method is less efficient than using an AER scheme (not shown). However, for the Ethernet based simulations, the opposite result is found for most cases (not shown). In these instances the cost of choosing either AER or Bit-packing depends entirely on the hardware platform. Alternatively, the hybrid method can be tuned, in some cases automatically, to perform on any of the hardware platforms tested.

### 4.3. Model complexity

The conclusions of this work are based on the idea that the neuron and synaptic computations are completed relatively quickly. Additionally, it is assumed that a single compute node can process a large number of neurons. This is the case for most point neuron implementations but as the complexity of the neuron model increases past the capabilities of the compute hardware, the time spent in numerical integration correspondingly increases. In these instances we suggest that optimization efforts be focused on improving the performance of numerical techniques.

### Conflict of interest statement

The authors declare that the research was conducted in the absence of any commercial or financial relationships that could be construed as a potential conflict of interest.
